# Effects of Different Application Methods of Potassium Dihydrogen Phosphate on the Growth, Cadmium Accumulation, and Translocation in Ramie (*Boehmeria nivea* L.) Under Cadmium Stress

**DOI:** 10.3390/plants15213478

**Published:** 2026-11-06

**Authors:** Jue Zhao, Shuo Zhang, Si He, Qinxu Song, Nicheng Wu, Hucheng Xing

**Affiliations:** 1 Ramie Research Institute, Hunan Agricultural University, Changsha 410128, China; zhaojue66zj@163.com (J.Z.); zs15145819770@163.com (S.Z.); hesiacademic@163.com (S.H.); songqinxu28@163.com (Q.S.); 15274735745@163.com (N.W.); 2 Yuelushan Laboratory, Changsha 410128, China; 3 Hunan Engineering Research Center for Germplasm Innovation and Utilization of Forage Crops, Changsha 410128, China

**Keywords:** ramie (*Boehmeria nivea* L.), cadmium stress, antioxidant defense system, subcellular distribution, cell wall retention, foliar nutrition, phytoextraction efficiency

## Abstract

Agronomic regulation is vital for improving the phytoextraction efficiency of economic crops in cadmium (Cd)-contaminated soils. Here, a two-season pot experiment evaluated the effects of foliar versus soil KH_2_PO_4_ applications on ramie (*Boehmeria nivea* L.) under Cd stress. Growth and phytoextraction performance peaked during the second harvest season. Foliar application of 0.2% KH_2_PO_4_ increased ramet number by 118.2% and alleviated oxidative damage by sequentially activating SOD, POD, and CAT. Subcellular analysis indicated that foliar KH_2_PO_4_ enhanced Cd sequestration within root cell walls, restricting excessive Cd translocation to photosynthetic tissues. In contrast, high-dose soil application exacerbated toxicity and inhibited biomass accumulation. Structural equation modeling showed that shoot biomass primarily drove total Cd extraction (β = 0.925), whereas stem cell wall Cd retention served as the major limiting factor (β = −0.471). Overall, low-dose foliar KH_2_PO_4_ provides a promising agronomic approach to enhance Cd phytoextraction by maximizing shoot biomass while limiting tissue toxicity.

## 1. Introduction

Cadmium (Cd) is a non-essential and highly toxic heavy metal that represents a severe abiotic stressor in agro-ecosystems. With intensive industrial and anthropogenic activities, the long-term accumulation of Cd in soils not only compromises soil biological health but also severely threatens plant growth and human food safety [1,2]. In China, approximately 19.4% of agricultural soils suffer from heavy metal contamination, with Cd being the predominant pollutant accounting for over 7% of arable areas [3]. Once absorbed by plant roots, Cd ions disrupt essential physiological processes, including nutrient uptake, stomatal conductance, and photosynthetic pigment synthesis. Furthermore, Cd triggers the excessive production of reactive oxygen species (ROS), causing severe membrane lipid peroxidation, enzyme inactivation, and organelle dysfunction [4,5,6]. Consequently, elucidating the physiological defense mechanisms and exploring sustainable agronomic interventions to alleviate Cd phytotoxicity have become critical frontiers in plant stress biology.

Phytoremediation using non-food, high-biomass economic crops has emerged as a promising and eco-friendly phytomanagement strategy [7,8,9,10]. While classic hyperaccumulators possess exceptional metal accumulation capabilities, their broad-scale agronomic deployment is severely hindered by slow growth rates, miniature biomass, and negligible economic value [11]. In this context, ramie (*Boehmeria nivea* L.), a perennial bast fiber crop native to China, has garnered extensive attention due to its massive biomass potential, well-developed rhizome systems, strong environmental adaptability, inherent heavy metal tolerance, and significant socioeconomic contribution as a vital textile raw material [12,13,14,15]. Ramie can maintain robust vegetative growth under moderate-to-high Cd stress and exhibits substantial shoot Cd accumulation capacity, achieving an annual metal extraction efficiency of up to 2.55% [16]. Nevertheless, excessive intracellular Cd accumulation inevitably impedes ramie vigor and metabolic homeostasis. Thus, developing targeted physiological and agronomic strategies to enhance ramie stress tolerance while maximizing its metal extraction efficiency remains a pressing task.

Exogenous nutrient supplementation, particularly with phosphorus (P) and potassium (K), represents an efficient approach to alleviate abiotic stress and regulate heavy metal dynamics in plants. Particularly, phosphorus regulates complex signaling pathways and metabolic processes to enhance plant tolerance against abiotic stresses [17]. Phosphorus is an indispensable macronutrient that drives cellular energy metabolism (ATP), nucleic acid biosynthesis, and membrane lipid integrity. Under heavy metal stress, P mitigates Cd phytotoxicity through coordinated extracellular and intracellular pathways. Extracellularly, phosphate ions precipitate with Cd^2+^ in the rhizosphere, decreasing the bioavailability of free Cd [18,19]. Intracellularly, P stimulates the synthesis of cell wall polysaccharides (e.g., pectin and hemicellulose), fortifying the cell wall retention barrier and sequestering Cd into vacuoles, which effectively shields metabolically active organelles (e.g., chloroplasts and mitochondria) from metal poisoning [20]. Concurrently, potassium (K^+^) acts as a pivotal osmotic regulator and enzymatic activator, maintaining cytosolic ion homeostasis and stomatal functioning. Adequate P-K nutrition comprehensively primes the plant antioxidant defense cascade and preserves the photosynthetic apparatus under stress conditions [21,22]. Therefore, potassium dihydrogen phosphate (KH_2_PO_4_), comprising both vital macronutrients, provides dual benefits of nutritional supplementation and stress mitigation, serving as an optimal agent to modulate plant responses to Cd stress.

In agronomic practice, foliar spraying and soil application represent two fundamentally different spatial intervention pathways with distinct physiological uptake modes. Foliar application provides a “top–down” targeted delivery directly to shoot tissues, circumventing rhizosphere immobilization, rapidly repairing damaged photosynthetic machinery, and potentially coordinating systemic root stress signals via source-sink transport [23,24,25]. In contrast, soil application functions as a “bottom–up” rhizosphere intervention, primarily establishing an initial barrier against root transmembrane influx through chemical immobilization and competitive ion antagonism at the root–soil interface [26,27]. Prior studies have indicated that foliar KH_2_PO_4_ improves shoot Cd extraction in Festuca arundinacea, whereas soil P application tends to intercept Cd within wheat root systems, hindering its upward translocation [28,29]. However, comparative studies examining how these two application pathways modulate plant performance in perennial fiber crops remain scarce. In particular, the divergent mechanisms of KH_2_PO_4_ application modes across multi-scale biological dimensions—spanning morphological allocation, photosynthetic pigment maintenance, dynamic antioxidant enzyme evolution, and subcellular cell wall retention—remain incompletely understood.

To address these knowledge gaps, this study employed the high-accumulating ramie cultivar ‘Xiangzhu 3′ in a consecutive two-season (first and second harvests) pot trial. We systematically established foliar spray and soil application treatments across concentration gradients of KH_2_PO_4_ (0.2%, 0.4%, and 0.6%) under Cd stress. The specific objectives were to: (1) investigate the differential impacts of foliar versus soil KH_2_PO_4_ applications on ramie biomass allocation, photosynthetic pigment integrity, the sequential activation of the antioxidant defense system (SOD, POD, CAT), and subcellular Cd distribution across cell walls, organelles, and soluble fractions; and (2) construct structural equation models (SEMs) to decipher the integrated causal regulatory pathways linking plant morphological phenotypes, physiological responses, subcellular partitioning, and macro-level Cd accumulation. This study aims to uncover the physiological and cellular mechanisms underlying KH_2_PO_4_-mediated Cd tolerance in ramie, offering practical insights into nutrient-assisted stress alleviation and sustainable crop management in heavy metal-contaminated lands. Although pot experiments have inherent limitations, they provide an essential regulated condition to validate fundamental mechanisms before extrapolating these inorganic amendment responses to complex field environments.

## 2. Results

### 2.1. Effects of Different KH_2_PO_4_ Application Methods on Agronomic Traits of Ramie Under Cd Stress

As shown by the three-way ANOVA results (Table S1), the growing season had highly significant independent effects on plant height (*p* = 0.009) and the number of ramets (*p* < 0.001). Furthermore, the interaction between the application method and KH_2_PO_4_ concentration (M×C) significantly affected plant height (*p* < 0.001), while the interaction between season and concentration (S×C) significantly regulated the number of ramets (*p* < 0.001).

**Figure 1:**
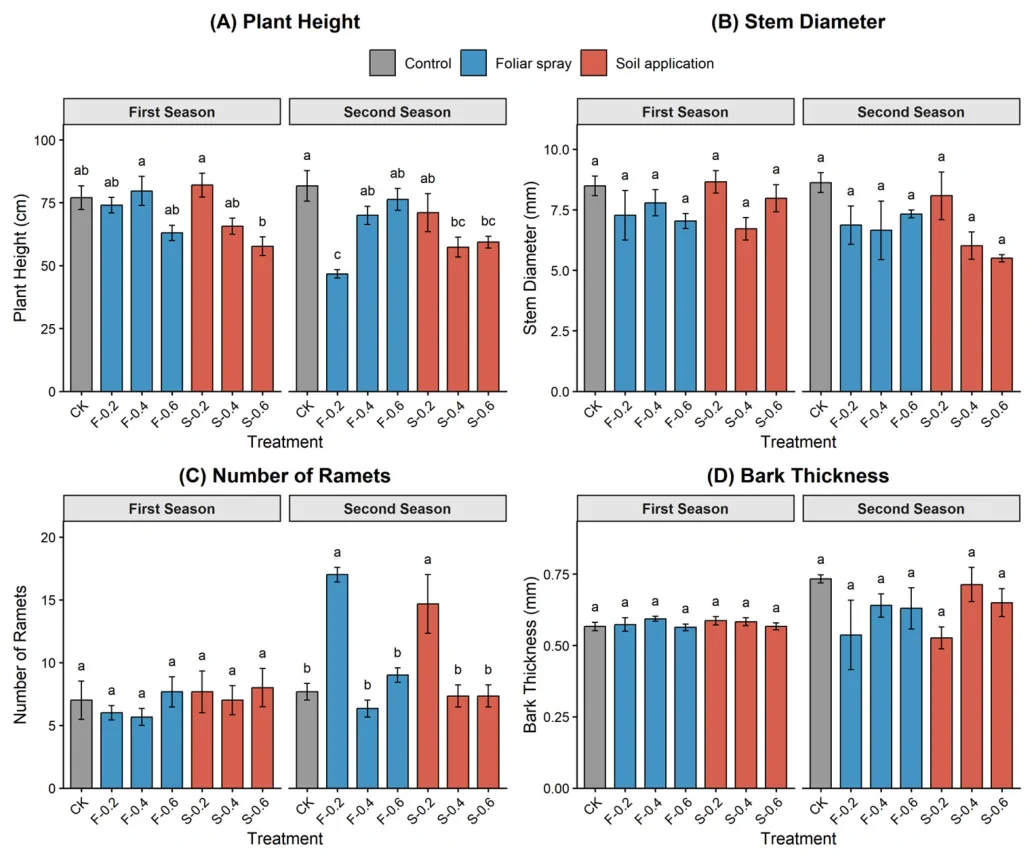
Effects of different treatments on the agronomic traits of ramie. (**A**) Plant height; (**B**) stem diameter; (**C**) number of ramets; (**D**) bark thickness. CK: Control; F-0.2, F-0.4, F-0.6: Foliar application of 0.2%, 0.4%, and 0.6% KH_2_PO_4_, respectively; S-0.2, S-0.4, S-0.6: Soil application of 0.2%, 0.4%, and 0.6% KH_2_PO_4_, respectively. Different lowercase letters indicate significant differences among treatments (*p* < 0.05, Tukey’s HSD test). The same applies below.

As illustrated in Figure 1, the overall plant height of ramie in the second harvest season was significantly greater than that in the first harvest season. In the first harvest season, high-concentration foliar application (F-0.6) significantly inhibited longitudinal plant growth, with its plant height (58.3 cm) being significantly lower than that of the control group (77.0 cm). In contrast, the soil application treatments had no significant effect on plant height in either season. No statistically significant differences were observed in stem diameter and bark thickness among the treatments. Notably, the stimulatory effect of the treatments on the number of ramets was particularly pronounced in the second harvest season: the low-concentration foliar application (F-0.2) significantly promoted lateral bud sprouting, causing the number of ramets (16.8 per pot) to surge by 118.2% compared with the concurrent control (7.7 per pot). Overall, a low-concentration foliar application (0.2%) can effectively stimulate the tillering potential of ramie, whereas a high-concentration foliar intervention (0.6%) may induce toxic inhibition of canopy growth.

### 2.2. Effects of Different KH_2_PO_4_ Application Methods on the Yield and Biomass of Ramie Under Cd Stress

According to Table S1, the seasonal shift exerted a decisive impact on both shoot and root dry weights (*p* < 0.001). Meanwhile, the two-way interactions between season and application method (S×M), and season and concentration (S×C) exerted highly significant regulatory effects on shoot dry weight.

**Figure 2:**
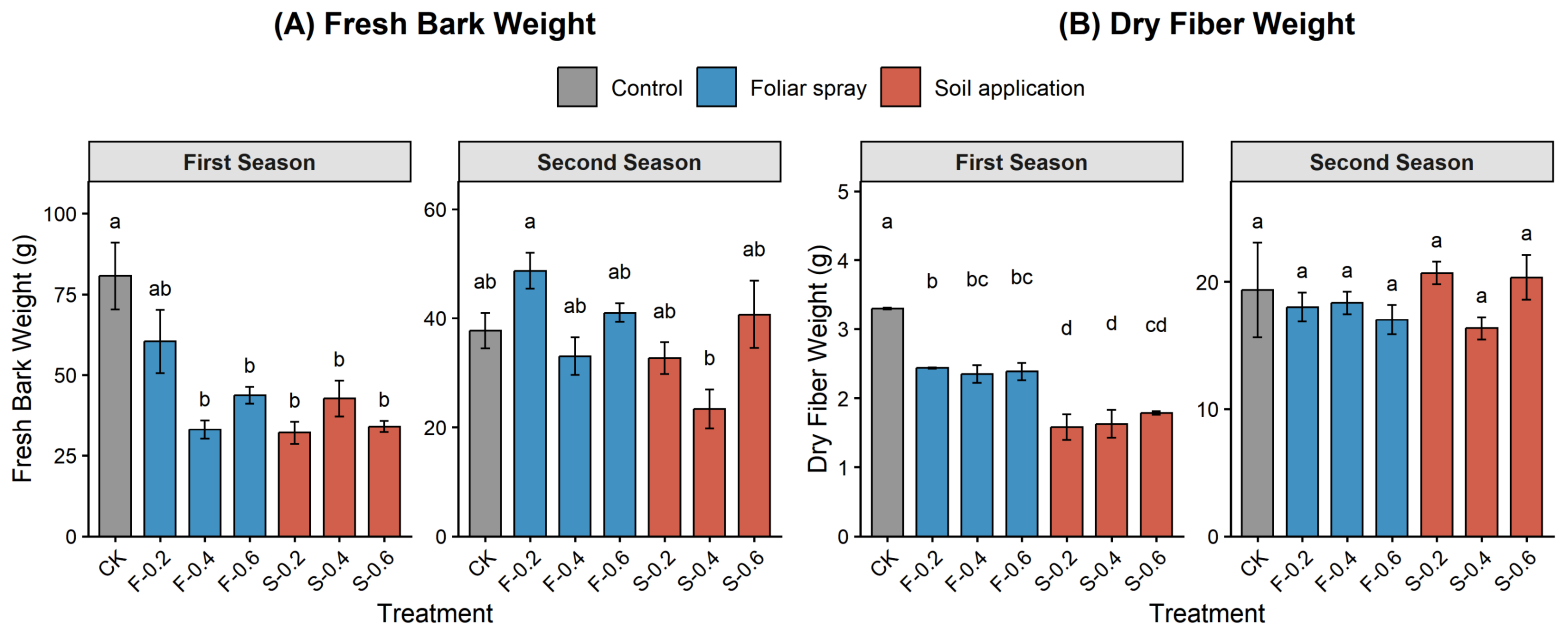
Effects of different treatments on the yield traits of ramie. (**A**) Fresh bark weight; (**B**) dry fiber weight. Different lowercase letters indicate significant differences between treatments at the *p* < 0.05 level.

Regarding yield traits (Figure 2), the fresh bark weight and dry fiber weight of the control group (CK) in the first harvest season were generally significantly higher than those in most KH_2_PO_4_ treatment groups. In the second harvest season, the fresh bark weight of the F-0.2 treatment reached the seasonal peak (~48 g), but the differences in dry fiber weight among the treatments tended to converge.

**Figure 3:**
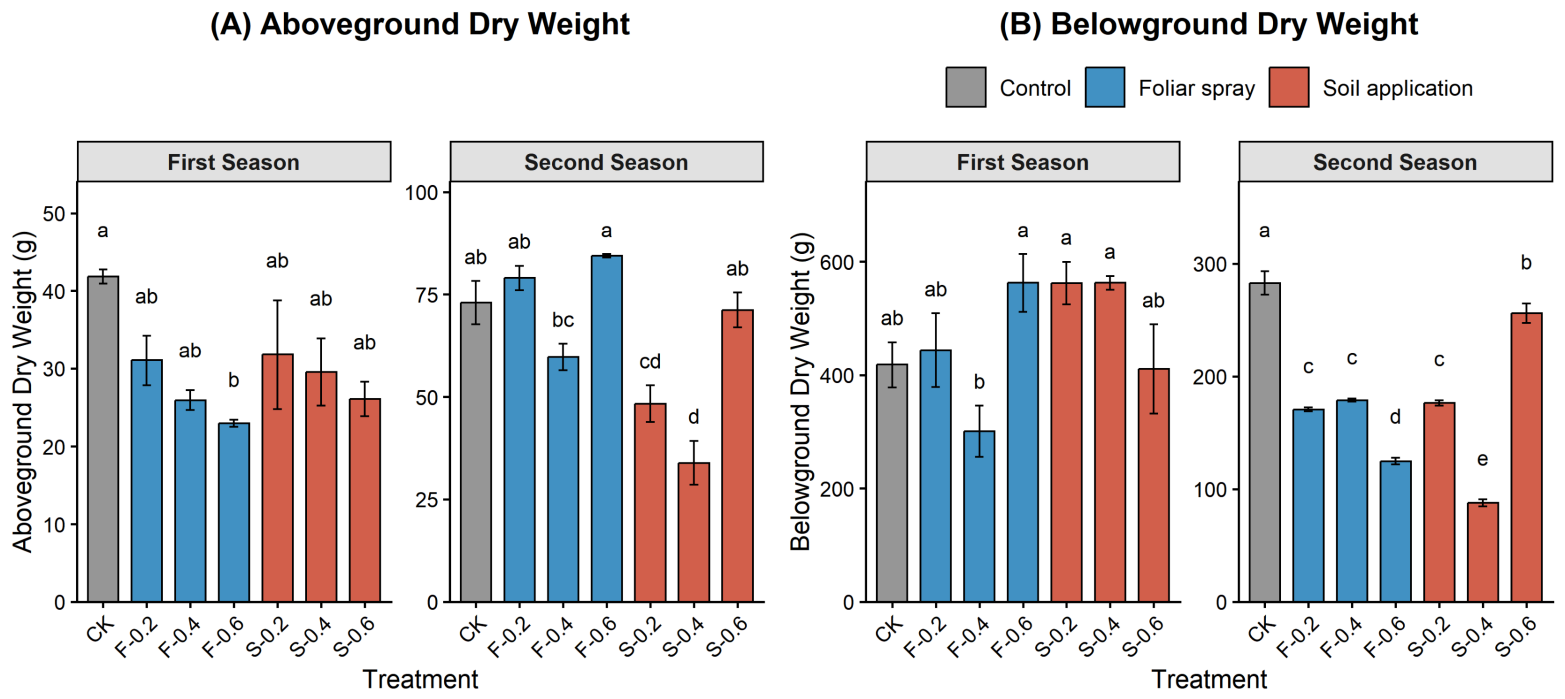
Effects of different treatments on the biomass of ramie. (**A**) Shoot dry weight; (**B**) root dry weight. Different lowercase letters indicate significant differences between treatments at the *p* < 0.05 level.

In terms of spatial biomass allocation (Figure 3), a strong seasonal dependency was exhibited among the treatments. Overall, the shoot dry weight in the second harvest season was significantly higher than that in the first harvest season, while the root dry weight showed an opposite trend. In the first harvest season, exogenous P intervention did not demonstrate a significant growth-promoting effect on the shoots (the F-0.6 treatment even caused the shoot dry weight to significantly decrease to the lowest level); however, in the roots, the F-0.6, S-0.2, and S-0.4 treatments all exhibited significant root-promoting effects. Entering the vigorously growing second harvest season, the effects of different application strategies diverged drastically: foliar application (especially F-0.6) showed a slight promotional trend on shoot biomass; conversely, all soil application treatments produced severe toxic inhibitory effects on both shoot and root growth. Specifically, the S-0.4 treatment caused the shoot and root dry weights to plummet by over 50% compared with the control. This indicates that in the second harvest season, which is characterized by high temperatures and humidity, the direct soil application of KH_2_PO_4_ might have exacerbated the combined salt and heavy metal stress within the rhizosphere microenvironment.

### 2.3. Effects of Different KH_2_PO_4_ Application Methods on Photosynthesis, Antioxidant Enzyme Activities, and Osmotic Adjustment Substances in Ramie Under Cd Stress

The three-way ANOVA revealed that the third-order interaction of season, application method, and concentration (S×M×C) exerted highly significant effects on MDA, SOD, POD, CAT, and osmotic adjustment substances (soluble sugars and proteins) (*p* < 0.01).

**Figure 4:**
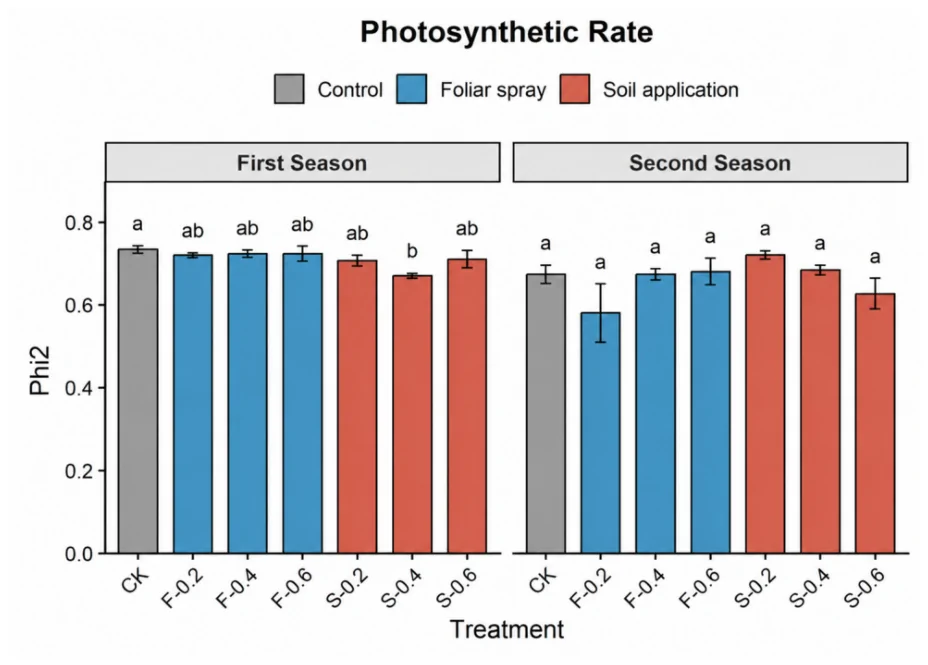
Effects of different treatments on the actual photochemical efficiency (Phi2) of ramie. Different lowercase letters indicate significant differences between treatments at the *p* < 0.05 level.

As illustrated in Figure 4, the effects of KH_2_PO_4_ on the actual photochemical efficiency of PSII (Phi2) tended to stabilize chronologically. During the first harvest season, moderate-to-high-concentration soil application (S-0.4) triggered a significant decline in Phi2; however, by the second harvest season, no significant differences in Phi2 were observed among the treatments, indicating that the photosynthetic system of ramie had developed a physiological adaptation to the long-term combined environment of heavy metals and exogenous P.

**Figure 5:**
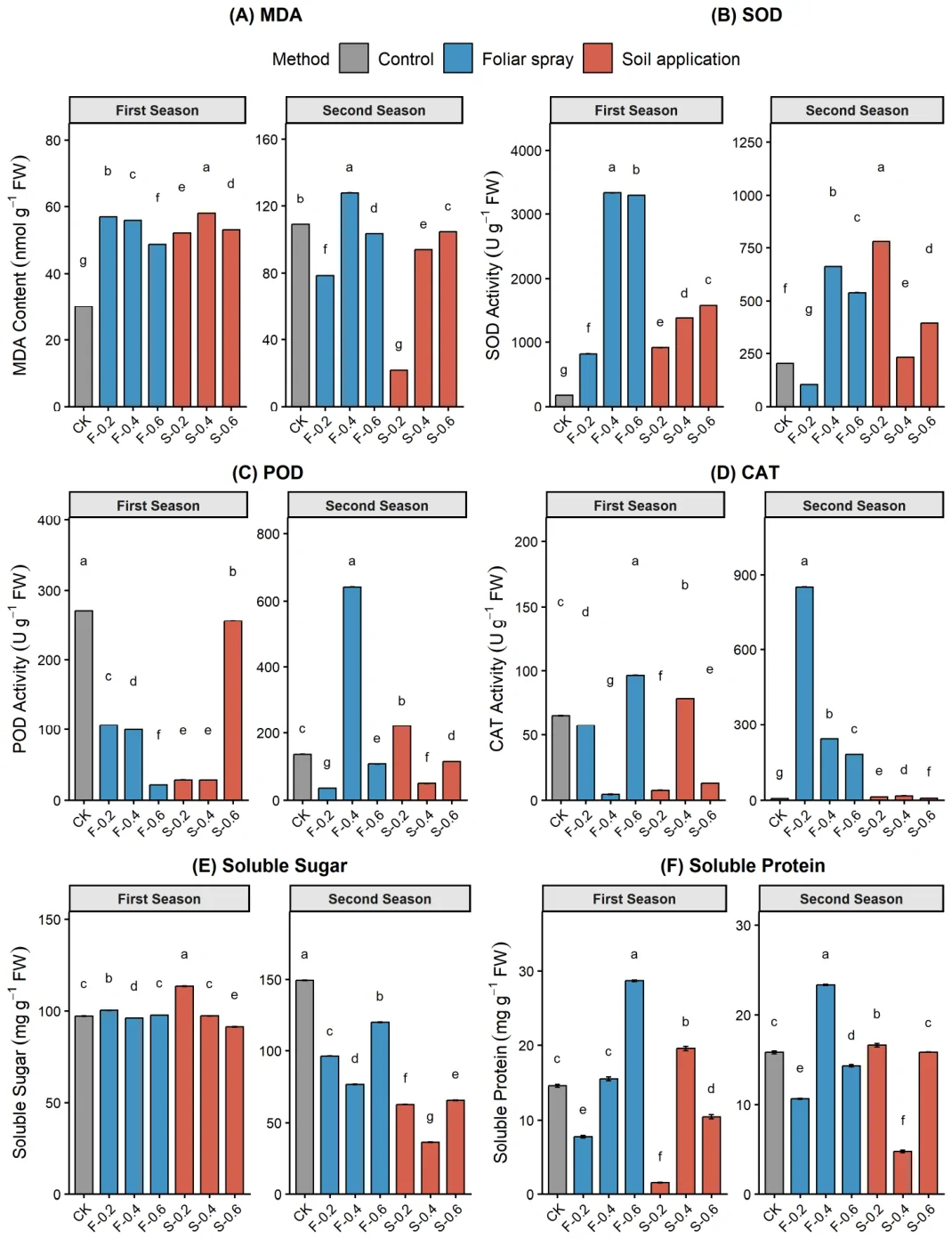
Effects of different treatments on antioxidant enzyme activities and osmotic adjustment substances of ramie. (**A**) Malondialdehyde (MDA) content; (**B**) superoxide dismutase (SOD) activity; (**C**) peroxidase (POD) activity; (**D**) catalase (CAT) activity; (**E**) soluble sugar content; (**F**) soluble protein content. Different lowercase letters indicate significant differences between treatments at the *p* < 0.05 level.

The antioxidant defense network of ramie exhibited a prominent characteristic of “temporal remodeling” (Figure 5A–D). In the first harvest season, all KH_2_PO_4_ treatments exacerbated membrane lipid peroxidation to some extent (characterized by an MDA surge). At this stage, the plants relied primarily on SOD for initial defense, with moderate-to-high-concentration foliar applications (F-0.4 and F-0.6) highly significantly inducing SOD activity. Entering the second harvest season, the overall SOD activity dropped substantially, and the detoxification mechanism shifted toward POD and CAT: F-0.4 highly significantly stimulated POD activity, whereas F-0.2 significantly upregulated CAT activity. Notably, the S-0.2 treatment in the second harvest season demonstrated an excellent cytomembrane protective effect, dropping the MDA content to an extremely low level while maintaining a relatively high SOD activity. Regarding osmotic regulation (Figure 5E,F), foliar spraying had a sustained and robust promotional effect on the synthesis of soluble proteins (peaking at F-0.6 in the first season and F-0.4 in the second season). Conversely, all KH_2_PO_4_ treatments in the second harvest season generally reduced the accumulation of soluble sugars.

### 2.4. Effects of Different KH_2_PO_4_ Application Methods on Tissue Distribution and Cell Wall Retention of Cd in Ramie Under Cd Stress

The interception of Cd by various tissues and cell walls of ramie exhibited a high degree of site specificity and strategic divergence (Table S1).

**Figure 6:**
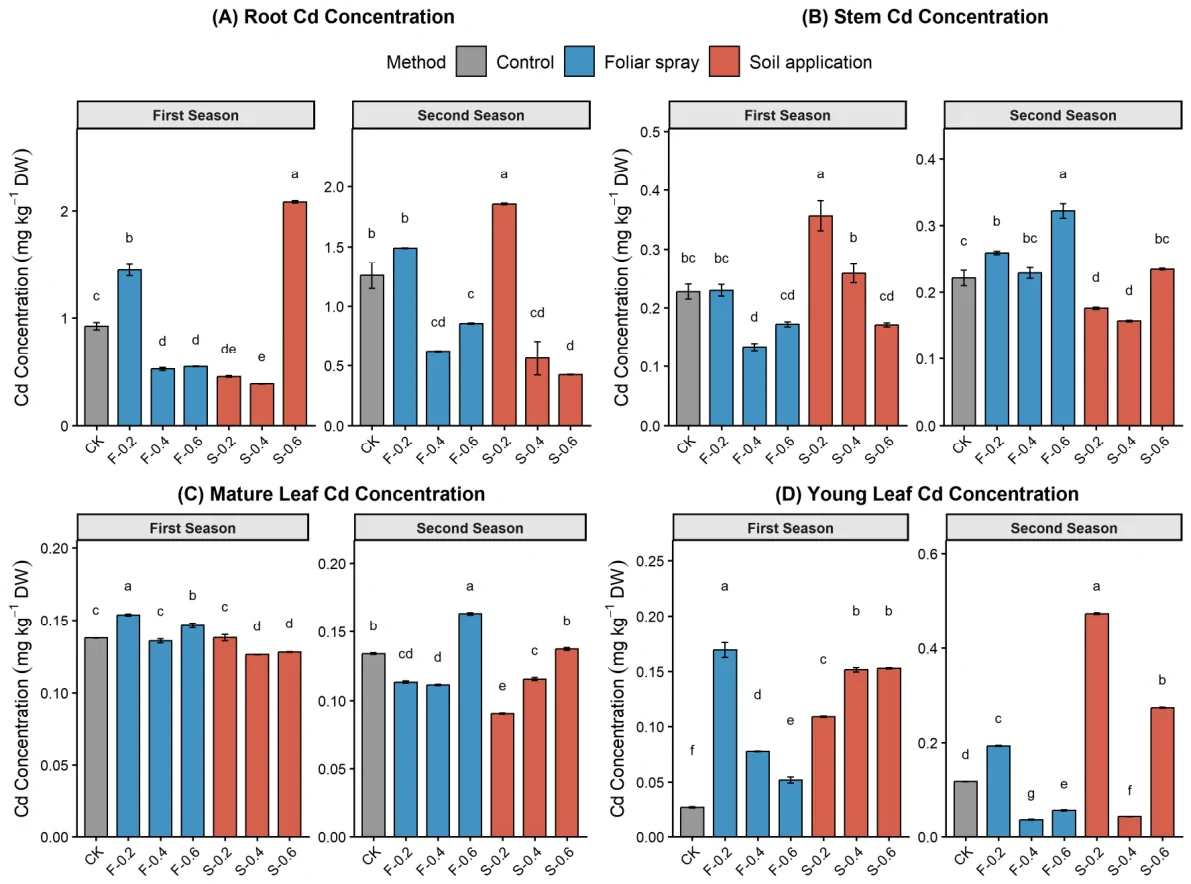
Effects of different treatments on tissue Cd contents in ramie. (**A**) Root Cd content; (**B**) stem Cd content; (**C**) mature leaf Cd content; (**D**) young leaf Cd content. Different lowercase letters indicate significant differences between treatments at the *p* < 0.05 level.

At the macroscopic tissue level (Figure 6), high-concentration soil application in the first harvest season significantly fortified the “Cd sink” effect of the roots. Under the S-0.6 treatment, the Cd content in young roots exceeded 2.0 mg·kg^−1^ DW, which was highly significantly greater than that in the control and foliar treatment groups. Concurrently, S-0.2 caused the Cd accumulation in stems to peak. In contrast, moderate-to-high-concentration foliar applications (F-0.4 and F-0.6) effectively suppressed the translocation of Cd to mature and young leaves. The divergence became more drastic in the second harvest season: F-0.4 exhibited an outstanding protective effect on photosynthetic tissues, suppressing the Cd content in both mature and young leaves to the lowest levels across the entire season. Conversely, S-0.2 significantly drove the upward translocation of Cd to nascent roots and young leaves, leading to a sharp surge in its toxic accumulation.

**Figure 7:**
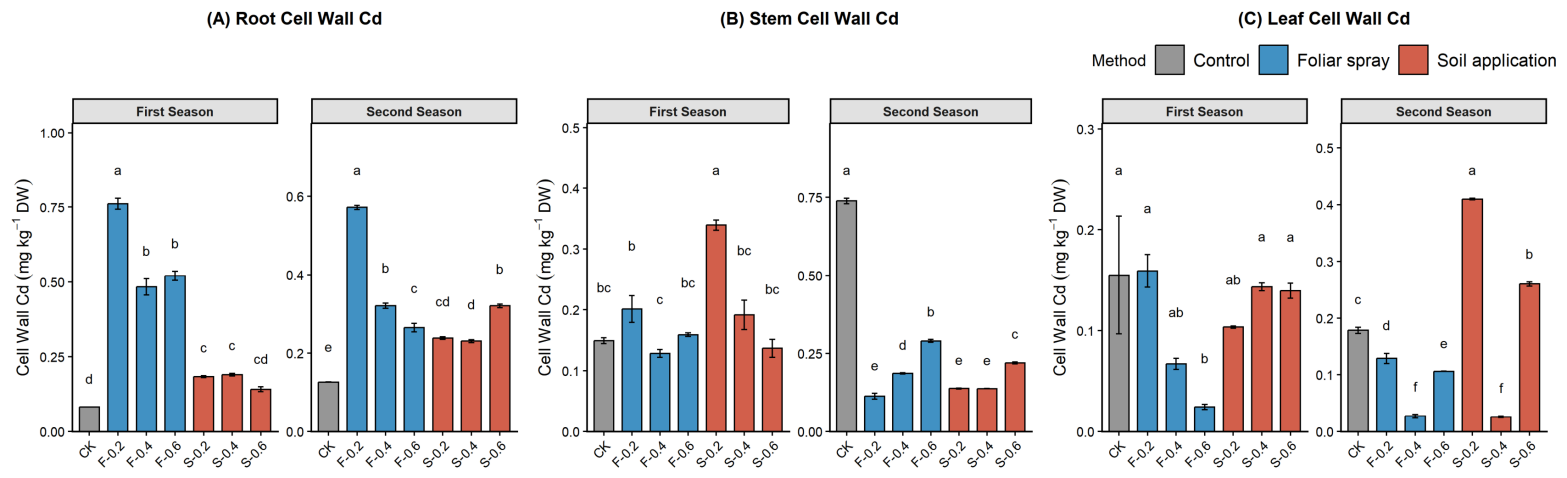
Effects of different treatments on cell wall Cd contents in ramie. (**A**) Root cell wall Cd content; (**B**) stem cell wall Cd content; (**C**) leaf cell wall Cd content. Different lowercase letters indicate significant differences between treatments at the *p* < 0.05 level.

Cell wall retention at the microscopic subcellular level further unveiled the underlying mechanisms of the aforementioned macroscopic distribution (Figure 7). At the root barrier front, F-0.2 significantly induced the interception of Cd by the root cell wall in both seasons. This microscopic retention effectively explains how foliar treatments block the upward translocation of Cd. In the shoots, the stem cell walls of the control group (CK) in the second harvest season accumulated extremely high levels of Cd (0.71 mg·kg^−1^ DW), whereas the S-0.2 treatment caused an ectopic surge of Cd in the leaf cell walls. Conversely, the F-0.4 treatment substantially unloaded the Cd burden from both stem and leaf cell walls. The macroscopic and microscopic data corroborated each other: precise foliar spraying (e.g., F-0.4) can effectively protect vulnerable young leaves by reinforcing the first line of defense in the root cell wall. In contrast, although direct soil application increased root interception, it was accompanied by a higher risk of heavy metal accumulation in active photosynthetic regions.

### 2.5. Effects of Different KH_2_PO_4_ Application Methods on Cd Bioconcentration Factor, Translocation Factor, and Accumulation in Ramie Under Cd Stress

**Figure 8:**
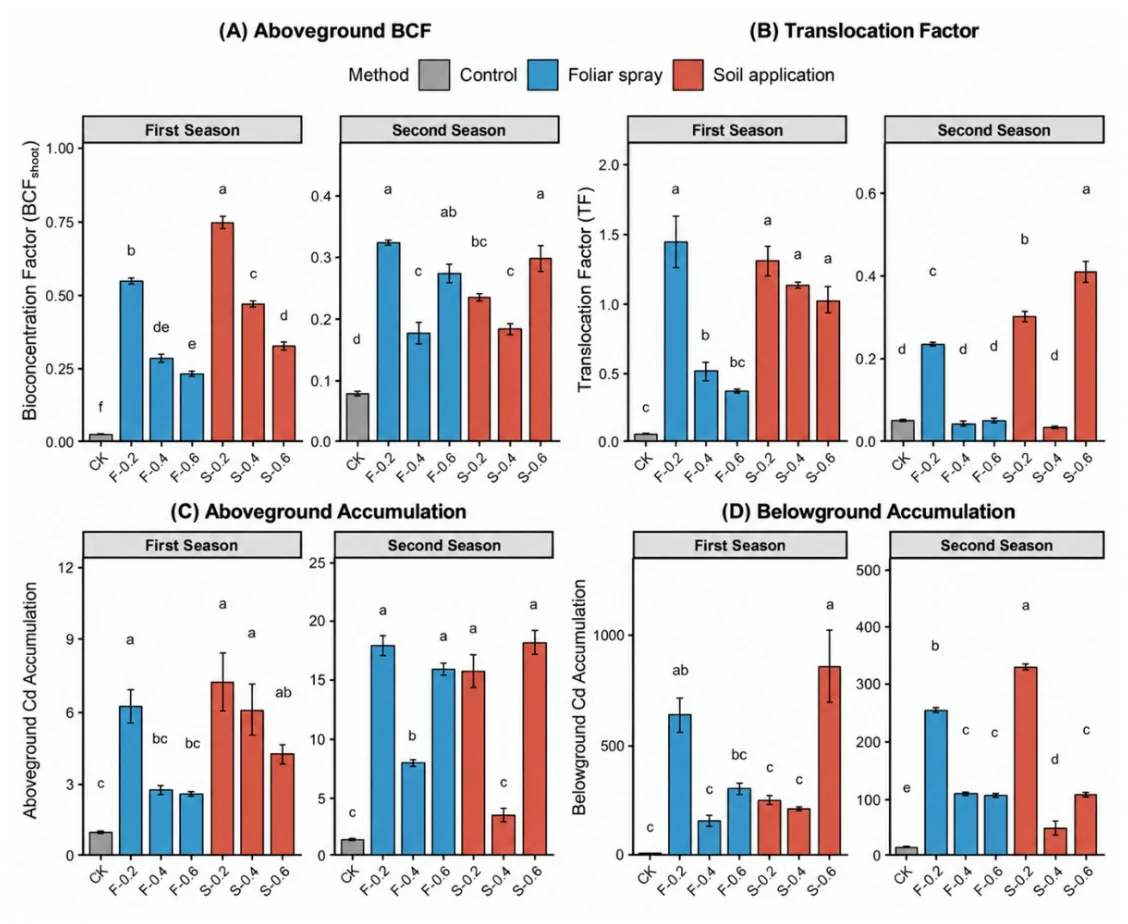
Effects of different treatments on Cd bioconcentration, translocation, and accumulation in ramie. (**A**) Shoot bioconcentration factor (BCF); (**B**) translocation factor (TF); (**C**) shoot Cd accumulation; (**D**) root Cd accumulation. Different lowercase letters indicate significant differences between treatments at the *p* < 0.05 level.

The bioconcentration factor (BCF) and translocation factor (TF) directly determine the engineering potential of phytoremediation (Figure 8). Compared with the control, exogenous KH_2_PO_4_ generally and significantly enhanced the extraction efficiency of ramie.

In the first harvest season, the shoot BCF under the S-0.2 treatment reached the highest level (0.76). Simultaneously, F-0.2 substantially broke the root retention limitation, with its TF surging to the seasonal peak (1.44). Entering the second harvest season, the translocation strategy underwent a reversal: foliar treatments (F-0.2 to F-0.6) significantly restricted the upward translocation of Cd (the TF dropping to 0.23–0.26), whereas the high-concentration soil treatment (S-0.6) maintained the highest translocation efficiency of the season (TF = 0.41).

The absolute accumulation based on biomass experienced an order-of-magnitude leap, with the mean shoot Cd accumulation in the second harvest season (17.84 µg/plant) far exceeding that in the first harvest season (5.61 µg/plant). In the pivotal second harvest season, F-0.2 exhibited the optimal absolute phytoextraction potential in shoots, while S-0.6 also performed excellently. Comprehensive analysis indicates that low-concentration foliar application (F-0.2) is the optimal agronomic approach to maximize “phytoextraction,” as it achieves maximized systemic detoxification and extraction while ensuring an immense shoot biomass. Conversely, soil application tends to amplify the sink potential of the roots, making it more suitable for in situ “phytostabilization” in severely contaminated areas.

### 2.6. Correlation of Indicators, PCA, and Structural Equation Modeling

**Figure 9:**
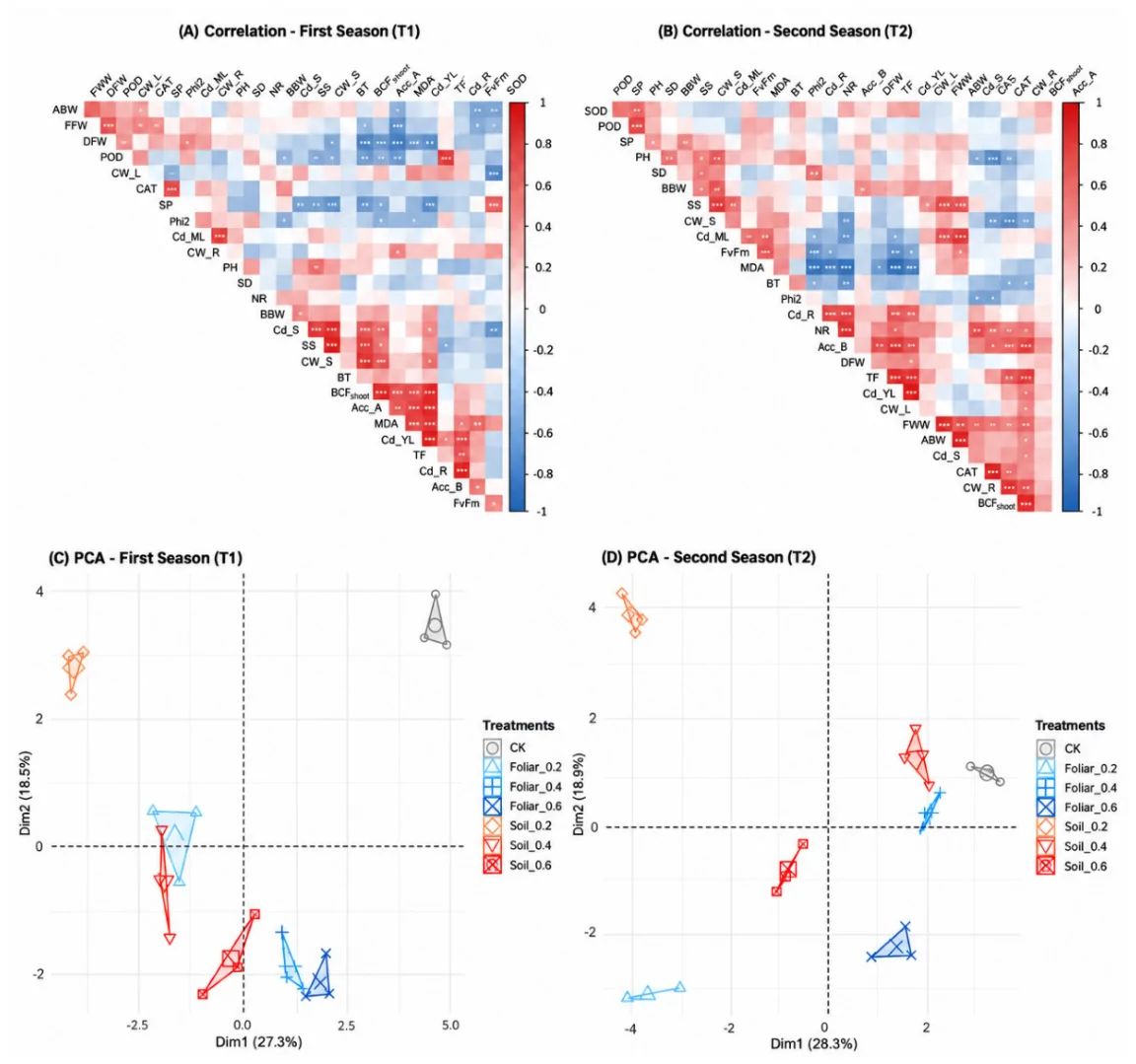
Correlation heatmaps and principal component analysis (PCA) for various indicators in the first harvest season (T1) and second harvest season (T2). (**A**) Correlation analysis in T1; (**B**) correlation analysis in T2; (**C**) PCA in T1; (**D**) PCA in T2. * *p* < 0.05, ** *p* < 0.01, *** *p* < 0.001.

The correlation heatmaps (Figure 9A,B) revealed that shoot biomass (ABW) was extremely and consistently positively correlated with shoot Cd accumulation (AccA) in both seasons (first harvest season: r = 0.85; second harvest season: r = 0.82). Meanwhile, MDA (a biomarker of lipid peroxidation) exhibited a sustained antagonistic stress effect on the actual photochemical efficiency (Phi2). The PCA (Figure 9C,D) further demonstrated a high dispersion of treatments in the principal component space during the first harvest season. However, during the second harvest season, as plant tolerance was established, the spatial distribution of phenotypes tended to converge, with PC1 being primarily driven by accumulation and translocation traits (BCF, TF, and AccA).

**Figure 10:**
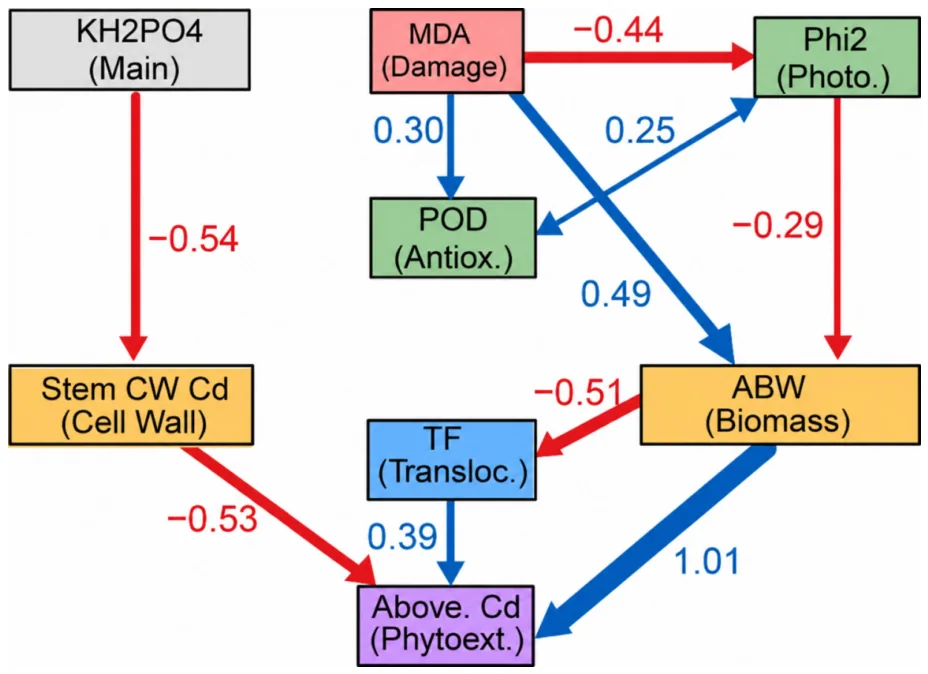
Path analysis of KH2PO4 regulating Cd accumulation in ramie based on structural equation modeling (SEM). Conc_std: Standardized main effect of KH2PO4 concentration; Inter_std: Standardized interactive effect of application method and concentration; MDA_std: Standardized malondialdehyde; POD_std: Standardized peroxidase; Phi2_std: Standardized actual photochemical efficiency (Phi2); CW_S_std: Standardized stem cell wall Cd content; ABW_std: Standardized shoot biomass; TF_std: Standardized translocation factor; AccA_std: Standardized shoot Cd accumulation. Red arrows indicate negative effects, while blue arrows indicate positive effects. The values adjacent to the paths represent standardized path coefficients.

To systematically decipher the latent causal network of cross-scale Cd accumulation regulation by KH_2_PO_4_, a structural equation model (SEM) with an excellent goodness-of-fit was constructed (Figure 10; χ^2^/df = 1.24, CFI = 0.956, RMSEA = 0.027). The path verification revealed the following: (1) An increase in exogenous concentration directly and negatively weakened the retention barrier of the stem cell wall (β = −0.404, *p* = 0.011). (2) At the physiological defense level, although MDA significantly inhibited photochemical efficiency Phi2 (β = −0.468, *p* = 0.001), it simultaneously positively stimulated POD activity (β = 0.305) and showed a strong positive association with biomass (β = 0.480, *p* < 0.001), revealing a complex mechanism of “stress-induced compensatory growth” under heavy metal stress. (3) Regarding the ultimate extraction target, shoot biomass acted as the absolute core dominant factor dictating shoot Cd accumulation (β = 0.925, *p* < 0.001). The translocation factor (TF) provided a secondary positive drive (β = 0.361, *p* < 0.001), whereas interception by the stem cell wall exerted a critical negative limiting role (β = −0.471, *p* < 0.001). In summary, the heavy metal remediation efficiency of ramie is essentially the outcome of a precise spatiotemporal synergy among source-end biomass expansion, longitudinal translocation efficiency, and subcellular retention barriers.

## 3. Discussion

### 3.1. Seasonal and Spatial Regulatory Mechanisms of KH_2_PO_4_ on Ramie Growth

In this study, the shoot/root biomass and agronomic traits of ramie in the second harvest season were significantly superior to those in the first harvest season. In conventional agricultural production, the first harvest of ramie usually exhibits better growth than the second harvest due to a longer growing period (approximately 3 months vs. 2 months). However, in the present pot experiment system, the first harvest represented the initial recovery growth stage following the “stumping and rootstock hilling” during the preceding winter. At this stage, the young root system was not yet fully constructed and was encountering intense soil Cd stress for the first time, leading to severely hindered early development. Entering the second harvest season, with the gradual establishment of the perennial rootstock system, physiological adaptation, and superior light and temperature conditions, the plants exhibited robust compensatory growth and carbon assimilation capacities.

Notably, high-concentration (0.6%) foliar application of KH_2_PO_4_ exerted a significant inhibitory effect on plant height during the first harvest season. The underlying mechanisms of this phenomenon may include: First, high-concentration exogenous salt ions induced localized osmotic stress on the leaf surface, restricting cell elongation. Second, excessive phosphate entering the mesophyll cells might have generated synergistic or antagonistic transmembrane transport effects with free Cd, thereby exacerbating metabolic disorders during the vulnerable growth stage (first harvest season) [30]. Finally, high-concentration salts might have altered the phyllosphere microenvironment, triggering stomatal closure and impairing photosynthetic efficiency. In contrast, soil application of high-concentration KH_2_PO_4_ did not exhibit significant inhibition of plant height. This is primarily attributed to the fact that phosphate (PO_4_^3−^) readily undergoes chemical precipitation with Cd^2+^ in the soil buffer system, thereby attenuating the bioavailability and phytotoxicity of Cd at the source [19]. Furthermore, low-concentration (0.2%) foliar application significantly promoted the sprouting of ramets during the second harvest season. This confirms that an appropriate concentration of exogenous P can effectively participate in cell division and energy metabolism, thereby stimulating apical dominance and the physiological potential for lateral bud development [22].

### 3.2. Significance of the Dynamic Transition in Inter-Seasonal Antioxidant Defense Strategies

Under severe abiotic stress, the overproduction of reactive oxygen species (ROS) can cause severe oxidative damage; therefore, activating robust antioxidative defense systems is a universally recognized mechanism for crop survival and stress alleviation [31,32]. This study revealed a prominent “seasonal transition of antioxidant strategies” in ramie under Cd stress. In the first harvest season, plants primarily relied on SOD as the first line of defense to scavenge ROS. However, entering the second harvest season, the core of the defense shifted significantly toward POD and CAT. This spatiotemporal transition mechanism can be attributed to the following: First, during the first harvest season, the ambient temperature was relatively low, and the rate of ROS burst was moderate; therefore, the SOD-dominated dismutation reaction was sufficient to maintain redox homeostasis. In contrast, against the backdrop of high temperature, strong light, and a dramatic surge in biomass during the second harvest season, cellular metabolism (mitochondrial and chloroplast electron transport) became extremely vigorous. The massive accumulation of H_2_O_2_ forced the plants to upregulate POD and CAT for highly efficient downstream scavenging [33]. Second, correlation analysis showed that SOD primarily acted as an emergency response enzyme to Cd toxicity in the first season, whereas POD and CAT were highly coupled with biomass expansion in the second season, becoming deeply involved in safeguarding growth and metabolism.

Furthermore, the application methods profoundly influenced this defense system. Foliar application bypassed root interception, delivering P directly to the leaves, which rapidly induced the synthesis of SOD and soluble proteins during the first harvest season. In contrast, soil application relied on long-distance root-to-shoot transport, thereby triggering the systemic expression of resistance via POD and CAT across the entire plant during the second harvest season [34]. Notably, 0.4% foliar application maintained the lowest MDA levels in both seasons, highlighting the optimal efficacy of this concentration in anchoring cytomembrane lipid peroxidation and maintaining membrane integrity.

### 3.3. Targeted Interventions of Application Methods on Cd Uptake and Macroscopic Tissue Translocation

Foliar and soil applications exhibited diametrically opposed targeted regulations on the source-sink distribution of Cd within the plant. Foliar application (especially at 0.4% concentration) suppressed the Cd concentration in mature leaves to the lowest levels (0.13 and 0.11 mg·kg^−1^ DW) in both seasons. The underlying mechanisms may involve: (1) Exogenous P directly strengthened the cation exchange capacity of the leaf cell wall, firmly intercepting Cd^2+^ in the apoplast and blocking its transmembrane influx into the protoplast. (2) P nutrition improved photosynthetic carbon assimilation, generating abundant soluble sugars and organic acids that could form stable chelates with Cd in the intracellular soluble fractions, thereby achieving internal detoxification [35]. (3) Foliar P signals might have initiated basipetal signaling, downregulating the expression of plasma membrane Cd transporters (such as the NRAMP or HMA families) in the roots, thus limiting heavy metal loading at the source [36].

Conversely, soil application significantly exacerbated Cd accumulation in roots and stems. In the first harvest season, the S-0.2 treatment caused a 28.2% surge in root Cd content compared with the control. This was not only because P and Cd formed insoluble precipitates (similar to Cd_3_(PO_4_)_2_) in the rhizosphere microdomain; more importantly, while the roots actively absorbed phosphate to maintain growth, they inevitably ingested more Cd through carrier-mediated co-transport or ion channel competition mechanisms [37]. Additionally, the application of P fertilizers might have reshaped the rhizosphere microbial community, stimulating the proliferation of specific phosphate-solubilizing or Cd-activating bacteria, thereby indirectly magnifying the Cd sink capacity of the roots [12].

Based on the bioconcentration (BCF) and translocation (TF) characteristics, the F-0.2 treatment possessed the highest TF value (1.44) along with an excellent systemic accumulation in the shoots, representing the optimal solution for executing “phytoextraction.” In contrast, soil application (e.g., S-0.4) substantially reduced the TF, propelling Cd to be deeply anchored in the belowground parts, which more closely aligns with the “phytostabilization” strategy to guarantee the quality of the aboveground fibers.

### 3.4. Compartmentalization Mechanisms of Cell Wall Retention at the Subcellular Level

The cell wall constitutes the first physicochemical barrier for plants to resist heavy metal invasion. Its abundant polymers, such as pectin and hemicellulose, provide massive negative charge sites (e.g., carboxyl and hydroxyl groups) that retain Cd^2+^ through electrostatic adsorption or coordination complexation [38,39,40,41]. This study found that the F-0.2 treatment significantly increased the Cd content in the root cell wall during the first harvest season. This strongly proves that low-concentration exogenous P specifically stimulated the de novo synthesis and demethylation of pectin in the root cell wall, thereby massively expanding the extracellular Cd-binding sites [42,43,44]. However, high-concentration (0.6%) spraying induced negative feedback, indicating that excessive P disrupted the normal assembly of the cell wall network structure.

Notably, the S-0.2 soil application significantly increased the proportion of Cd in the stem cell wall. Research by Yang and Wang [30] on poplar confirmed that adequate P nutrition can significantly advance the development of the secondary xylem and the thickening of tracheid cell walls. In the present study, the continuous supply of P from the soil accelerated the lignification process of ramie stems, transforming the stem from merely mechanical support into a critical “sponge zone” that intercepts Cd transport to the leaves. Meanwhile, the leaf cell wall Cd content under the F-0.4 treatment was the lowest, which is perfectly consistent with its extremely low total leaf Cd, confirming that this treatment globally weakened the driving force for allocating Cd to the leaves.

### 3.5. The Core Causal Network of Cd Remediation Parsed by Structural Equation Modeling (SEM)

The SEM deeply decoded the hidden causal network underlying Cd accumulation driven by KH_2_PO_4_. First, shoot biomass contributed an absolutely dominant direct positive effect (β = 0.925) to the final amount of Cd extracted. The data thoroughly refute the mere “dilution effect” hypothesis, proving that in the ramie remediation system, the capacity expansion of the “biomass pump” (enrichment effect) far outweighs concentration dilution. The heavy metal extraction efficiency of ramie is absolutely driven by biomass, which is highly consistent with the accumulation characteristics of high-biomass crops proposed by Peng et al. [16] and by field phytoattenuation studies using high-biomass energy crops [45].

Second, the translocation factor (TF) provided the second-highest positive driving force (β = 0.361), confirming that unblocking the “root–stem–leaf” ascending pathway is the core target for enhancing remediation efficiency. It is known that hormones such as ethephon can fortify heavy metal translocation [46]. In this study, the coupling of exogenous P and hormones most likely intervened in the ethylene signaling pathway via a phosphorylation cascade, synergistically over-regulating the activities of transport pumps like HMAs [47,48]. More importantly, the stem cell wall Cd content exerted a strong negative effect on the total shoot accumulation (β = −0.471). This conclusively indicates that the more Cd is intercepted by the stem cell wall, the more restricted the total amount of Cd entering the harvestable tissues will be. Finally, the indirect effect path of MDA on biomass confirmed that oxidative damage is the most critical constraint hindering the transition from environmental stress to remediation efficiency. Maintaining a low MDA level to preserve photochemical efficiency (Phi2) is the prerequisite for ensuring that the plant sustains a massive biomass baseline.

### 3.6. Research Limitations and Prospects

As a mechanism exploration at the pot-experiment scale, this study has the advantage of high soil homogeneity, but it has not yet fully encompassed the interferences of high field heterogeneity (such as varied pH, organic matter abundance, and complex indigenous microbiomes). Future in situ field validations across different habitats in Cd-contaminated farmlands are urgently needed. Secondly, the current gradients of 0.2%, 0.4%, and 0.6% have outlined the dose–effect contours, but precise threshold inflection points may require further exploration; Response Surface Methodology (RSM) could be employed in the future for precision optimization [49]. In the molecular dimension, combined transcriptomics and spatial metabolomics technologies should be introduced in the future to meticulously map the co-localization and in tissue microdomains. Additionally, while ramie is a non-food fiber crop, caution must be exercised when extrapolating these findings to edible tuberous food crops, where Cd hyper-accumulation poses a severe, direct threat to human health.

## 4. Materials and Methods

### 4.1. Experimental Materials

The experimental plant material used was ‘Xiangzhu 3′ (*Boehmeria nivea* L.), a high-quality and high-yield ramie cultivar widely planted in southern China, provided by the Ramie Research Institute of Hunan Agricultural University. This cultivar exhibits physiological characteristics such as massive biomass, a well-developed root system, a strong perennial rootstock capacity, and extreme tolerance to heavy metal stress. The experimental soil was a typical paddy soil collected from the surface layer (0–20 cm) at the pot experiment site of Hunan Agricultural University. The experimental containers were standard white plastic pots (inner diameter 23 cm, height 25 cm). The exogenous agent potassium dihydrogen phosphate (KH2PO4) and all other chemical reagents used were of analytical grade (Sinopharm Chemical Reagent Co., Ltd., Shanghai, China).

### 4.2. Experimental Design

The pot experiment was conducted in an open-air net house at the Ramie Research Institute of Hunan Agricultural University. In August 2021, air-dried and sieved soil was placed into the pots. Exogenous Cd (applied as a CdCl_2_ solution) was added to achieve a target concentration of 25 mg/kg. The soil was thoroughly mixed, maintained at field water capacity, and aged for one month to simulate a realistic Cd-contaminated microenvironment. Healthy ramie cuttings with uniform growth (approximate plant height of 30 cm) were selected. After their roots were washed free of soil, they were transplanted into the pots, with three uniform seedlings retained per pot. In December 2021, the ramie plants were subjected to stumping (cutting the stems) and rootstock hilling management, allowing them to overwinter naturally.

A completely randomized block design was adopted, comprising seven treatments: (1) CK: Control (single Cd stress without KH_2_PO_4_ application); (2) F-0.2: Foliar application of 0.2% KH_2_PO_4_; (3) F-0.4: Foliar application of 0.4% KH_2_PO_4_; (4) F-0.6: Foliar application of 0.6% KH_2_PO_4_; (5) S-0.2: Soil application of 0.2% KH_2_PO_4_; (6) S-0.4: Soil application of 0.4% KH_2_PO_4_; and (7) S-0.6: Soil application of 0.6% KH_2_PO_4_. Each treatment consisted of three replicates, totaling 21 pots.

The exogenous KH_2_PO_4_ interventions were carried out over two consecutive main growing seasons in the following year.

First harvest season (March to early June 2022): The initial chemical treatment was applied during the vigorous growth stage. For foliar application, both the adaxial and abaxial surfaces of the leaves were evenly sprayed with approximately 100 mL of the respective KH_2_PO_4_ solution per pot until just before runoff (no obvious dripping). For soil application, an equal volume of the solution was uniformly irrigated into the topsoil of the rhizosphere. The treatments were applied every 10 days for a total of three applications, followed by the harvest and stumping of the first season’s crop.

Second harvest season (mid-June to early August 2022): Relying on the newly sprouted tillers from the perennial rootstocks, the aforementioned treatment procedures were repeated (applied three times), and sampling was conducted at the mature stage of the second harvest.

### 4.3. Measurements and Analytical Methods

#### 4.3.1. Agronomic Traits and Biomass

At the harvest stages of both seasons, plant height, stem diameter, and the number of ramets were measured in situ. After peeling, the bark thickness in the middle section was measured using a vernier caliper, and fresh bark weight and dry fiber weight were recorded. Subsequently, the plants were separated into shoots (stems and leaves) and roots, and thoroughly rinsed with deionized water. The samples were heated at 105 °C for 30 min to inactivate enzymes, and then dried to a constant weight in a thermostatic oven at 75 °C. The dry weights of the shoots and roots were recorded separately. For subsequent chemical and physiological analyses, the three plants within each pot were pooled as a single composite biological replicate to minimize individual variations.

#### 4.3.2. Photosynthesis and Physiological Parameters

Plants with uniform growth were selected. The actual photochemical efficiency of PSII (Phi2) and maximum photochemical quantum yield of PSII (Fv/Fm) of the fifth fully expanded functional leaf from the apex were measured in vivo using a MultispeQ multifunctional photosynthesis analyzer (Photosynq Inc., East Lansing, MI, USA) between 9:00 and 11:00 AM on clear days. Each leaf was measured five times, and the average value was recorded.

Fresh leaves were ground in an ice bath, and the enzyme solution was extracted by centrifugation for physiological and biochemical assays. Malondialdehyde (MDA) content was determined using the thiobarbituric acid (TBA) method. For the antioxidant enzyme system, superoxide dismutase (SOD) activity was measured using the nitroblue tetrazolium (NBT) photochemical reduction method; peroxidase (POD) activity was assayed via the guaiacol method; and catalase (CAT) activity was determined using UV spectrophotometry. Regarding osmotic adjustment substances, soluble sugar (SS) and soluble protein (SP) contents were determined using the anthrone colorimetric method and the Coomassie Brilliant Blue G-250 method, respectively.

#### 4.3.3. Determination of Cd Content

Total Cd: The dried plant organ samples were pulverized and passed through a 100-mesh nylon sieve. Accurately weighed 0.20 g samples were placed into digestion tubes and cold-digested overnight with a mixture of HNO_3_-HClO_4_ (*v*/*v*, 4:1). Subsequently, they were digested in a graphite digestion block with a programmed temperature increase until the solution became colorless and transparent. After the digestion solution reached a constant volume, the Cd concentration was determined using a flame atomic absorption spectrophotometer (FAAS, TAS-990superF, Beijing Persee, Beijing, China). Strict quality control was maintained during the measurement by simultaneously using national standard reference materials (GBW) and reagent blanks, with the Cd recovery rate stabilizing between 95% and 105%.

Cell wall Cd: Fresh root, stem, and leaf tissues were accurately weighed and homogenized in an ice bath with a pre-cooled extraction buffer (containing 250 mM sucrose, 50 mM Tris-HCl, and 1 mM dithiothreitol, pH 7.5). The homogenate was centrifuged at 3000× *g* for 15 min at 4 °C, and the resulting pellet represented the crude cell wall fraction. The pellet was purified by repeatedly washing it three times with the extraction buffer and then dried. The Cd content in the cell wall was determined using the mixed-acid digestion method described above.

#### 4.3.4. Calculation of Cd Bioconcentration and Translocation Factors

In the context of phytoextraction, evaluating the bioconcentration capacity of the harvestable aboveground tissues (shoots) is essential for determining the actual remediation potential of crops [50]. Accordingly, the extraction and translocation efficiency of Cd by the plants were evaluated using the following equations:

Bioconcentration Factor (BCF) = Cd content in shoots (mg/kg)/Total Cd content in soil (mg/kg)

Translocation Factor (TF) = Cd content in shoots (mg/kg)/Cd content in roots (mg/kg)

Cd Accumulation (µg/plant) = Tissue Cd content (mg/kg) × Corresponding tissue dry biomass (g)

### 4.4. Statistical Analysis

All experimental data are expressed as “Mean ± Standard Error (SE)”. Assuming the data met the assumptions of normality and homogeneity of variance, a three-way ANOVA was performed to evaluate the independent and interactive effects of the growing season (Season), application method (Method), and KH_2_PO_4_ concentration (Concentration) on various parameters. When significant differences were found among treatments, Tukey’s HSD test was applied for post hoc multiple comparisons (α = 0.05). Pearson correlation coefficients were used to evaluate the correlations among physiological and biochemical variables, biomass, and Cd accumulation. Based on the multivariate analysis results, structural equation modeling (SEM) was constructed using the lavaan package in R to quantitatively decipher the multidimensional latent path mechanisms through which KH_2_PO_4_ regulates macroscopic Cd accumulation in ramie. All statistical analyses and data visualizations were completed using packages such as ggplot2 within the R 4.5.1 platform (R Foundation for Statistical Computing, Vienna, Austria).

## 5. Conclusions

In conclusion, this study deciphers the multi-scale physiological and subcellular mechanisms by which differential KH_2_PO_4_ application regimes modulate ramie (*Boehmeria nivea* L.) tolerance and Cd accumulation across consecutive growing seasons. Ramie exhibited pronounced seasonal developmental plasticity; the vigorous vegetative regrowth during the second harvest season served as the primary biological driver for maximizing shoot biomass and metal phytoextraction capacity.

Exogenous KH_2_PO_4_ applications triggered divergent physiological adaptation strategies depending on the application route. Low-concentration foliar spraying (0.2%) applied during the vigorous growth stage significantly promoted lateral ramet outgrowth and alleviated Cd-induced abiotic stress. This was achieved by chronologically upregulating the antioxidant enzyme cascade (SOD, POD, and CAT) and preserving photosynthetic integrity. At the subcellular level, foliar KH_2_PO_4_ strategically fortified the cell wall retention barrier and facilitated intracellular soluble chelation in roots, thereby shielding vulnerable metabolic organelles (such as chloroplasts and mitochondria) from Cd toxicity and sustaining active shoot growth. In contrast, direct soil application imposed combined metal–salinity osmotic stress within the rhizosphere, which inhibited root vitality and biomass accumulation. Furthermore, structural equation modeling (SEM) confirmed that shoot biomass expansion was the predominant direct driver governing total Cd extraction (β = 0.925), whereas stem cell wall interception functioned as an essential regulatory checkpoint restricting excessive metal flux (β = −0.471).

Overall, stage-specific foliar supplementation of low-dose (0.2%) KH_2_PO_4_ during the peak vegetative stage represents an efficient and scalable plant-nutritional strategy. It synergistically optimizes abiotic stress tolerance, growth vigor, and heavy metal extraction efficiency, providing novel physiological insights and practical guidelines for nutrient-assisted crop management in heavy metal-affected agricultural ecosystems.

## Data Availability

Data are contained within the article.

## Supplementary Materials

The following supporting information can be downloaded at https://www.mdpi.com/article/10.3390/plants15213478/s1, Figure S1: Dynamic changes in cadmium (Cd) content in the lower mature leaves of ramie under foliar spraying and soil application treatments (at 0, 10, 20, and 30 d after foliar application); Figure S2: Dynamic changes in antioxidant enzyme activities and osmoregulatory substances in ramie under foliar spraying and soil application treatments (at 0, 10, 20, and 30 d after foliar application); Figure S3: Dynamic changes in photosynthetic parameters in ramie under foliar spraying and soil application treatments (at 0, 10, 20, and 30 d after foliar application); Figure S4: Dynamic changes in plant height and stem diameter of ramie under foliar spraying and soil application treatments (at 0, 10, 20, and 30 d after foliar application); Table S1: Three-way ANOVA (PP-values) of the effects of season (S), application method (M), concentration (C), and their interactions on the agronomic, physiological, and Cd accumulation traits of ramie; Table S2: Unstandardized and standardized path coefficients, standard errors, and significance levels for the structural equation model (SEM) evaluating the mechanisms of Cd phytoextraction.
