# Supplementary material for: Effects of Different Application Methods of Potassium Dihydrogen Phosphate on the Growth, Cadmium Accumulation, and Translocation in Ramie (Boehmeria nivea L.) Under Cadmium Stress
Source: Plants (Basel). 2026 Nov 6;15(21):3478. doi: 10.3390/plants15213478 (PMC13766105; doi:10.3390/plants15213478)
Supplement: Supplementary file 1 [file plants-15-03478-s001.zip › plants-4663036-supplementary.pdf]

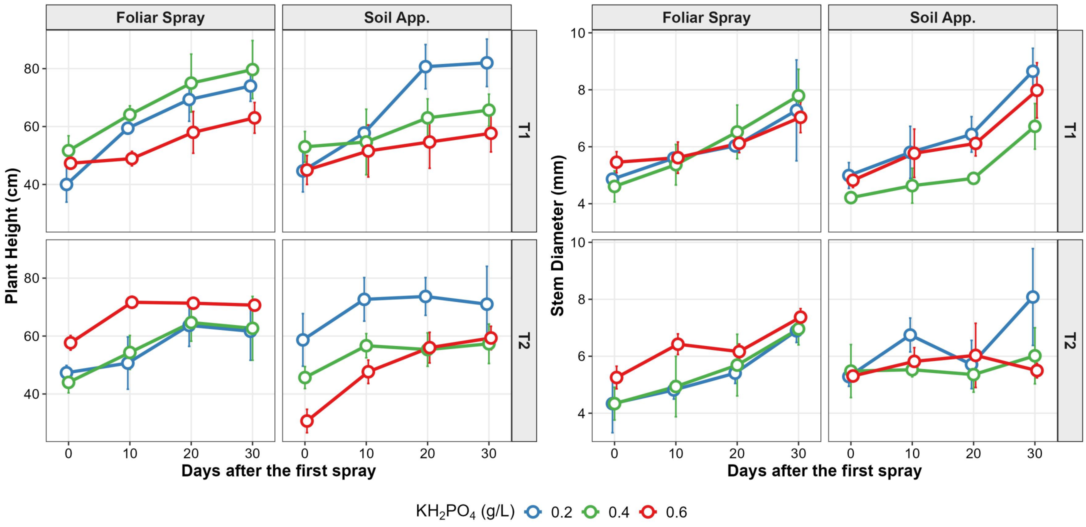

Figure S1. Dynamic changes in cadmium (Cd) content in the lower mature leaves of ramie under foliar spraying and soil application treatments (at 0, 10, 20, and 30 d after foliar [illegible]

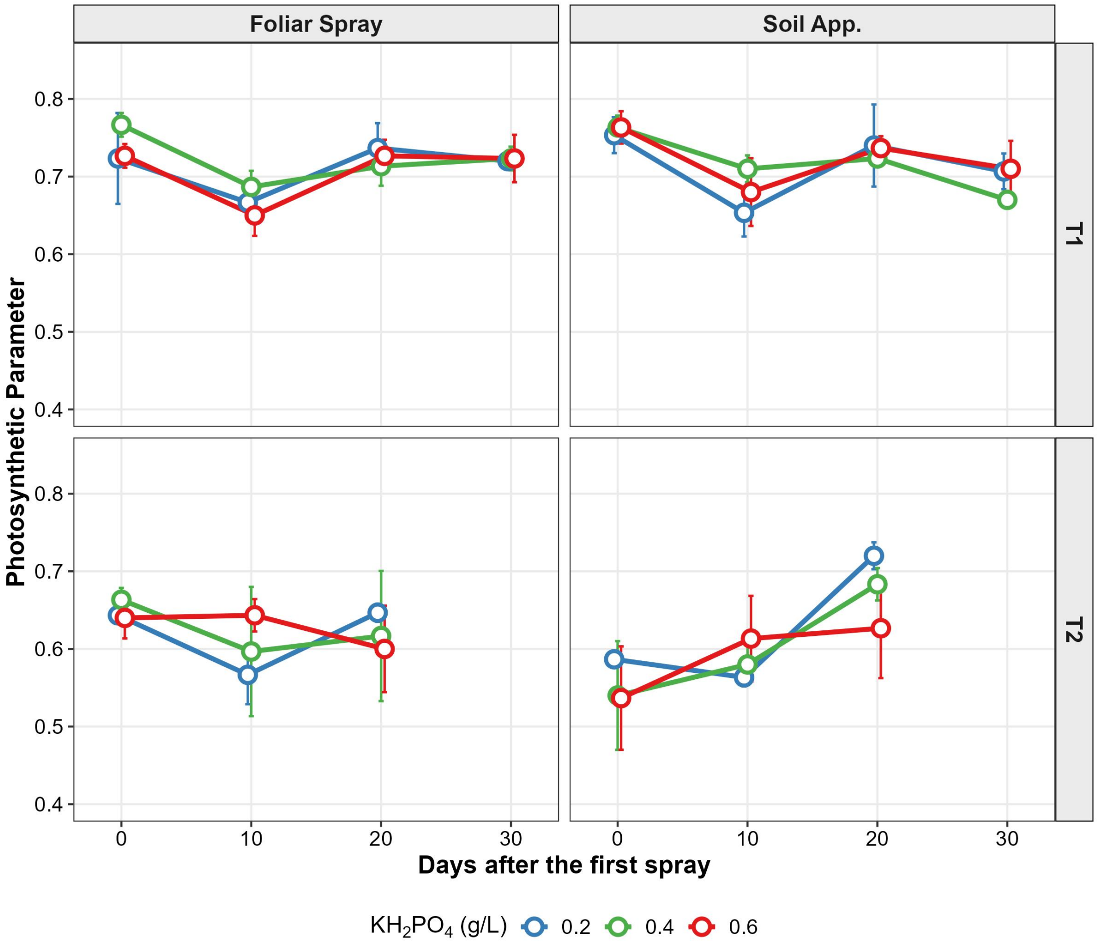

Figure S2. Dynamic changes in antioxidant enzyme activities and osmoregulatory substances in ramie under foliar spraying and soil application treatments (at 0, 10, 20, and 30 d after foliar application).

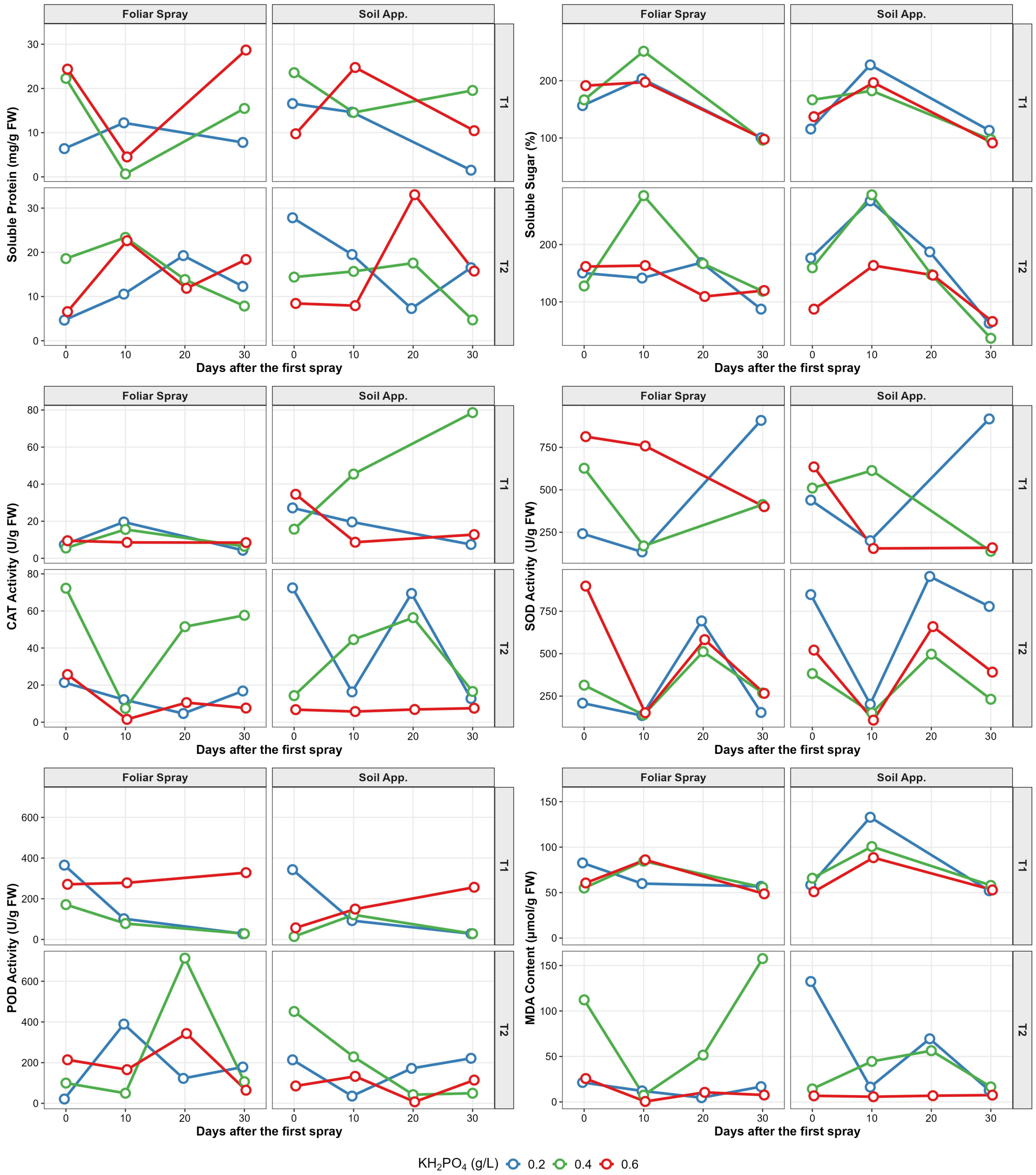

Figure S3. Dynamic changes in photosynthetic parameters in ramie under foliar spraying and soil application treatments (at 0, 10, 20, and 30 d after foliar application).

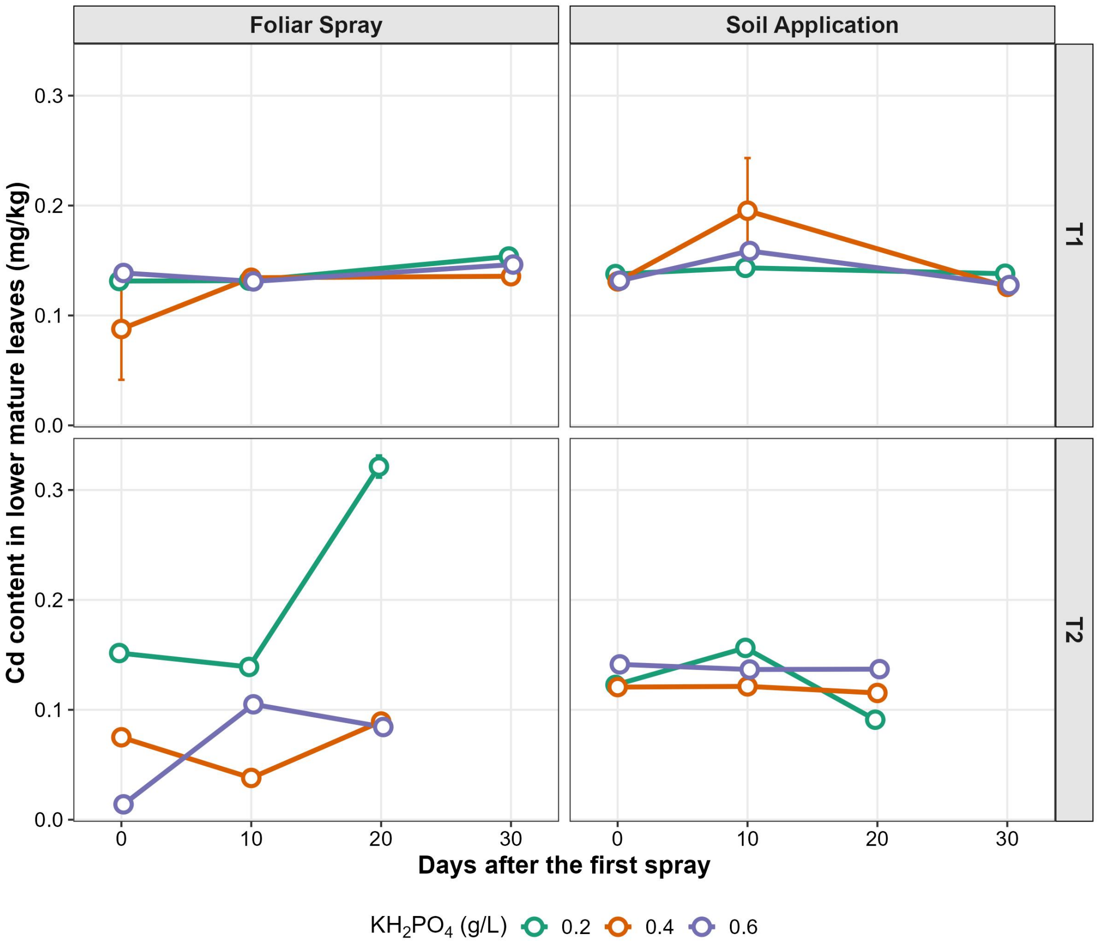

Figure S4. Dynamic changes in plant height and stem diameter of ramie under foliar spraying and soil application treatments (at 0, 10, 20, and 30 d after foliar application).

Table S1. Three-way ANOVA (PP-values) of the effects of season (S), application method (M), concentration (C), and their interactions on the agronomic, physiological, and Cd accumulation traits of ramie.

Variable	Description	S	M	C	SxM_p	SxC_p	MxC_p	SxMxC_p
PH	Plant height	0.0089**	0.2623 ns	0.2787 ns	0.6831 ns	0.0006***	0.0000***	0.0811 ns
SD	Stem diameter	0.0447*	0.9989 ns	0.1459 ns	0.3016 ns	0.8137 ns	0.0817 ns	0.2288 ns
NR	Number of ramets	0.0001***	0.9358 ns	0.0000***	0.1351 ns	0.0000***	0.5151 ns	0.5554 ns

ST	Bark thickness	0.2003 ns	0.6110 ns	0.1170 ns	0.6646 ns	0.1289 ns	0.9126 ns	0.7541 ns
DWF	Dry fiber weight	0.0000***	0.5306 ns	0.2278 ns	0.0415*	0.2520 ns	0.0630 ns	0.0652 ns
BA	Aboveground dry weight	0.0000***	0.0001***	0.0001***	0.0000***	0.0000***	0.1950 ns	0.3234 ns
BU	Belowground dry weight	0.0000***	0.0444*	0.0693 ns	0.1698 ns	0.7807 ns	0.1881 ns	0.0000***
Qy	Photosynthetic rate	0.0051**	0.8607 ns	0.9546 ns	0.0723 ns	0.4133 ns	0.0373*	0.0596 ns
FvFm	PSII quantum yield	0.1666 ns	0.0392*	0.0025**	0.0769 ns	0.0737 ns	0.6366 ns	0.3503 ns
MDA	Malondialdehyde	0.0000***	0.0000***	0.0000***	0.0000***	0.0000***	0.0000***	0.0000***
CAT	Catalase	0.0000***	0.0000***	0.0000***	0.0000***	0.0000***	0.0000***	0.0000***
POD	Peroxidase	0.0000***	0.0000***	0.0000***	0.0000***	0.0000***	0.0000***	0.0000***
SOD	Superoxide dismutase	0.0000***	0.0000***	0.0000***	0.0000***	0.0000***	0.0000***	0.0000***
CCML	Mature leaf Cd	0.0000***	0.0000***	0.0000***	0.8925 ns	0.0000***	0.0000***	0.0000***
CCYR	Young root Cd	0.0234*	0.0765 ns	0.0000***	0.0019**	0.0000***	0.0000***	0.0000***
CCYS	Young stem Cd	0.1359 ns	0.8163 ns	0.0000***	0.0000***	0.0000***	0.0001***	0.0004***
CCYL	Young leaf Cd	0.0000***	0.0000***	0.0000***	0.0000***	0.0000***	0.0000***	0.0000***
CCAP	Aboveground Cd content	0.0000***	0.0000***	0.0000***	0.0075**	0.0000***	0.0003***	0.0000***
CCUP	Belowground Cd content	0.0234*	0.0765 ns	0.0000***	0.0019**	0.0000***	0.0000***	0.0000***
CAAP	Aboveground Cd accumulation	0.0000***	0.5597 ns	0.0000***	0.0007***	0.0000***	0.0446*	0.0030**
CAUP	Belowground Cd accumulation	0.0000***	0.2137 ns	0.0000***	0.2637 ns	0.0001***	0.0000***	0.0000***
AEC	Aboveground enrichment coeff.	0.0000***	0.0000***	0.0000***	0.0000***	0.0000***	0.0894 ns	0.0000***
ATF	Aboveground translocation factor	0.0000***	0.0000***	0.0000***	0.0081**	0.0000***	0.0001***	0.0015**

Table S2. Unstandardized and standardized path coefficients, standard errors, and significance levels for the Structural Equation Model (SEM) evaluating the mechanisms of Cd phytoextraction.

**Dependent Variable**	**Independent Variable**	**Unstandardized Estimate**	**Standard Error (SE)**	**Z-value**	**P-value**	**Standardized Estimate (Beta)**	**Significance**
MDA_std	Conc_std	0.1728	0.1671	1.0336	0.3013	0.1728	ns
MDA_std	Inter_std	0.1426	0.1671	0.8533	0.3935	0.1426	ns
POD_std	Conc_std	-0.1545	0.1667	-0.927	0.3539	-0.1545	ns
POD_std	Inter_std	0.0814	0.166	0.4901	0.6241	0.0814	ns
POD_std	MDA_std	0.305	0.152	2.0069	0.0448	0.305	*
Pn_std	Conc_std	-0.0362	0.1555	-0.2329	0.8158	-0.0362	ns
Pn_std	Inter_std	0.1834	0.1549	1.1841	0.2364	0.1834	ns
Pn_std	MDA_std	-0.4675	0.1418	-3.2982	0.001	-0.4675	***
CW_S_std	POD_std	-0.1013	0.1426	-0.7102	0.4776	-0.1013	ns
CW_S_std	Conc_std	-0.4048	0.1599	-2.5319	0.0113	-0.4048	*
CW_S_std	Inter_std	0.0343	0.1602	0.2141	0.8305	0.0343	ns
ABW_std	Pn_std	-0.2858	0.1323	-2.1596	0.0308	-0.2858	*
ABW_std	POD_std	0.0345	0.1243	0.2776	0.7814	0.0345	ns
ABW_std	MDA_std	0.4797	0.1376	3.4869	0.0005	0.4797	***
TF_std	POD_std	-0.2135	0.1264	-1.688	0.0913	-0.2097	ns

				7			
TF_std	CW_S_std	0.0778	0.1253	0.6208	0.5347	0.0764	ns
TF_std	ABW_std	-0.5381	0.1263	-4.2618	0	-0.5286	***
AccA_std	ABW_std	1.0924	0.0872	12.5328	0	0.9252	***
AccA_std	TF_std	0.4184	0.0861	4.857	0	0.3608	***
AccA_std	CW_S_std	-0.5557	0.0726	-7.6589	0	-0.4706	***

Notes: Conc_std: Standardized main effect of KH2PO4 concentration;Inter_std: Standardized interaction effect between application method (foliar vs. soil) and KH2PO4 concentration;MDA_std: Malondialdehyde;POD_std: Peroxidase;Pn_std: Net photosynthetic rate;CW_S_std: Stem cell wall Cd content;ABW_std: Aboveground biomass (dry weight);TF_std: Translocation factor;AccA_std: Aboveground Cd accumulation.SE: Standard Error.Significance levels: *** $p < 0.001$; ** $p < 0.01$; * $p < 0.05$; ns: not significant ($p \geqslant 0.05$).
